# L-DOPA Neurotoxicity Is Mediated by Up-Regulation of DMT1−IRE Expression

**DOI:** 10.1371/journal.pone.0004593

**Published:** 2009-02-25

**Authors:** Fang Du, Zhong-ming Qian, Li Zhu, Xiao Mei Wu, Wing-ho Yung, Ting-yuk Tsim, Ya Ke

**Affiliations:** 1 Department of Physiology, Faculty of Medicine, The Chinese University of Hong Kong, New Territories, Hong Kong; 2 Department of Applied Biology & Chemical Technology, Hong Kong Polytechnic University, Kowloon, Hong Kong; 3 Department of Neurobiology and Jiangsu Key Laboratory of Neuroregeneration, Nantong University, Nantong, People's Republic of China; University of Giessen, Germany

## Abstract

**Background:**

The mechanisms underlying neurotoxicity caused by L-DOPA are not yet completely known. Based on recent findings, we speculated that the increased expression of divalent metal transporter 1 without iron-response element (DMT1−IRE) induced by L-DOPA might play a critical role in the development of L-DOPA neurotoxicity. To test this hypothesis, we investigated the effects of astrocyte-conditioned medium (ACM) and siRNA DMT-IRE on L-DOPA neurotoxicity in cortical neurons.

**Methods and Findings:**

We demonstrated that neurons treated with L-DOPA have a significant dose-dependent decrease in neuronal viability (MTT Assay) and increase in iron content (using a graphite furnace atomic absorption spectrophotometer), DMT1−IRE expression (Western blot analysis) and ferrous iron (55Fe(II)) uptake. Neurons incubated in ACM with or without L-DOPA had no significant differences in their morphology, Hoechst-33342 staining or viability. Also, ACM significantly inhibited the effects of L-DOPA on neuronal iron content as well as DMT1−IRE expression. In addition, we demonstrated that infection of neurons with siRNA DMT-IRE led to a significant decrease in DMT1−IRE expression as well as L-DOPA neurotoxicity.

**Conclusion:**

The up-regulation of DMT1−IRE and the increase in DMT1−IRE-mediated iron influx play a key role in L-DOPA neurotoxicity in cortical neurons.

## Introduction

Parkinson's disease (PD) is a progressive neurodegenerative disorder that affects approximately 1% of the people over the age of 60 [1]. This disorder is mainly characterized by the degeneration of dopamine-containing neurons in the substrantia nigra. This brain section is therefore deprived of adequate amounts of the neurotransmitter dopamine [2]. Because dopamine is unable to access the brain directly, L-3,4-dihydroxyphenylalanine (L-DOPA), its natural precursor, is used in clinical treatment of patients with PD. Until now, L-DOPA remains the most effective drug for the symptomatic control of PD [3], [4]. However, accumulated evidence shows that the therapeutic efficacy of L-DOPA is gradually lost over time, and abnormal involuntary movements, dyskinesias, gradually emerge as a prominent side effect of the previously beneficial doses of the drug [5]–[7].

The precise molecular mechanisms underlying the neurotoxicity caused by L-DOPA are not yet completely known. Available data suggest that L-DOPA might have the ability to significantly affect iron distribution in the brain. The changes in brain iron distribution induced by L-DOPA might be one of the causes of the neurotoxicity of L-DOPA. A clinical study [8] demonstrated that L-DOPA could significantly affect brain ceruloplasmin (CP), a major factor in the regulation of regional brain iron, and that L-DOPA-treated PD patients had a significantly higher CP than those who were not given L-DOPA. A pathological study of postmortem brain tissue showed that the levels of iron storage protein ferritin were significantly lower in several brain regions of PD patients treated with L-DOPA than those in the age-matched control patients [9]. In a recent study, we demonstrated that L-DOPA induces a significant increase in the expression of divalent metal transporter 1 without iron-response element (DMT1−IRE), but not divalent metal transporter 1 with iron-response element (DMT1+IRE), TfR1 or Fpn1, and a remarkable increase in ferrous uptake in cells [10].

Based on these findings, plus the potential role of DMT1−IRE in neuronal iron uptake and the implication of iron as a major generator of reactive oxygen species, we speculated that the upregulation of DMT1−IRE might play a critical role in the development of L-DOPA neurotoxicity. L-DOPA might have a role to increase DMT1−IRE expression, which in turn leads to a remarkable increase in DMT1−IRE-mediated ferrous iron uptake by neurons. Consequently, the increased ferrous iron in neurons generates highly reactive hydroxyl radicals via the Fenton reaction or Haber-Weiss reaction. In turn, these free radicals can damage the biological molecules of neurons, leading to the development of L-DOPA neurotoxicity. To test this hypothesis, we investigated the effects of astrocyte-conditioned medium (ACM) and siRNA DMT−IRE on L-DOPA neurotoxicity by observing the changes in morphology and Hoechst 33342 staining, measuring neuronal viability, neuronal iron content, expression of DMT1−IRE, DMT1+IRE, TfR1 and Fpn1 proteins and ferrous iron uptake in cortical neurons in the present study. Our results provide solid evidence that the upregulation of DMT1−IRE plays a key role in the development of L-DOPA neurotoxicity in vitro. The findings imply that inhibition of DMT1−IRE expression or neuronal iron uptake might be an effective approach to prevent or delay the development of L-DOPA neurotoxicity in PD patients.

## Materials and Methods

### Materials

Unless otherwise stated, all chemicals were obtained from Sigma Chemical Company, St. Louis, MO, USA. The scintillation cocktail and tubes were purchased from Beckman Coulter Company, Fullerton, CA, USA and ^55^FeCl_3_ from Perkinelmer Company, Wellesley, MA, USA. The antibodies against DMT1+IRE, DMT1−IRE and Fpn1 were purchased from Alpha Diagnostic International Company, San Antonio, TX, USA and mouse anti-rat TfR1 monoclonal antibody was obtained from BD Transduction Laboratories, BD Biosciences Pharmingen, USA. The specific antibodies against neuron microtubule-associated protein 2 (MAP2) and astrocyte glial fibrillary acadic protein (GFAP) were purchased from Chemicon International Ltd, UK and Hoechst 33342 from Polysciences Inc., Warrington, PA, USA.

### Primary cortical neurons

Primary cortical neurons were prepared from embryonic Day 18 (E18) rats as previously described [11] with minor modifications. The purity of these cultures was assessed by staining for the neuron specific antibodies against MAP2 and the astrocyte marker GFAP.

### Preparation of astrocytes culture medium

Primary astrocytes were prepared as previously described [12], [13]. The purity of astrocyte cultures was determined using anti-GFAP antibody. The medium was changed to DMEM/F12 supplemented with 2% newborn calf serum (NCS), collected 48 hours later (astrocyte conditioned medium, ACM), centrifuged at 3000 r/min for 15 min to remove cellular debris and then stored at −80°C.

### Experimental Design

To investigate the effects of L-DOPA on cortical neurons, the cells were treated with different concentrations of L-DOPA (0, 1, 5, 10, 100 or 200 µM) in DMEM+5%FBS for 16 hours and then morphological changes, neuronal viability, cell iron content, the expression of DMT1−IRE, DMT1+IRE, TfR1 and Fpn1 proteins and NTBI (non-transferrin bound iron) uptake were determined. To detect the effects of ACM on the neurotoxicity induced by L-DOPA, neurons were incubated with or without L-DOPA in DMEM+5%FBS or ACM for 16 hours followed by the relevant measurements. To further confirm whether the increased expression of DMT1−IRE was involved in the neurotoxicity induced by L-DOPA, neurons were pre-incubated with siRNA DMT1−IRE for 24 hours before treatment with L-DOPA.

### Morphological observation

Morphological changes of the neurons were observed under phase contrast microscopy or fluorescence microscopy.

### Determination of cell viability

The cell viability was determined using an MTT assay as described previously [14], [15]. Optical density (OD) was measured at 570 nm by the use of the ELX-800 microplate assay reader (Bio-tek, USA).

### Immunocytochemistry

Immunocytochemical experiments were performed using neuron specific mouse anti-MAP2, astrocyte specific anti-GFAP, rabbit anti-rat DMT1−IRE, DMT1+IRE, TfR1 and rabbit anti-mouse Fpn1 antibodies respectively [16]. Cortical neurons in slides were incubated with one of the above antibodies: anti-MAP2 (1∶200), anti-GFAP (1∶200), anti-rat DMT1−IRE (1∶500), DMT1+IRE (1∶500), TfR (1∶200) and anti-mouse Fpn1 (1∶500) for 24 hours, followed by FITC-labeled goat anti-mouse IgG (1∶200), or Rhodamine-labeled goat anti-rabbit IgG (1∶200) (Invitrogen, USA) as the secondary antibody. Fluorescent images were captured by using a Nikon Eclipse TE2000-U microscope (Nikon, UK) equipped with T-FL Epi-FI attachment and a SPOT cooled CCD camera (Diagnostic Instruments, INC, US). SPOT software version 4.6 was used for image acquisition (Diagnostic Instruments, INC, US).

### Hoechst 33342 staining

Hoechst 33342 staining was used to detect morphological features of apoptotic cell death. Cortical neurons pre-treated with or without different concentrations of L-DOPA were stained with Hoechst 33342. The cells were then examined under a fluorescent microscope. Undamaged cell nuclei were large and diffusely stained whereas apoptotic nuclei showed chromatin that was condensed and fragmented.

### Iron measurement

The slides were fixed with 4% paraformalin for 10 min. After three washes with PBS, the slides were incubated in Perl's solution containing 4% potassiumferrocanide and an equal volume of 1.2 mmol/L hydrochloride acid solution for 16 hours at 4°C and then washed with deionized water 3 times, followed by dehydration through 95% alcohol and mounting with xylene. Iron staining was observed under a Nikon Eclipse TE2000-U microscope (Nikon, UK). The total iron in the neurons was also determined using a graphite furnace atomic absorption spectrophotometer (GFAAS, Perkin Elmer, Analyst 100) as previously described [17], [18].

### Western blot analysis

Neurons that received different treatments were washed with ice-cold PBS, homogenized with lysis buffer and then subjected to sonication using Soniprep 150 (MSE Scientific Instruments, London, UK). After centrifugation at 10,000 g for 15 min at 4°C, the supernatant was collected, and protein content was determined using the Bradford assay kit (Bio-Rad). Aliquots of the cell extract containing 30 µg of protein were diluted in 2 µl sample buffer (50 mM Tris, pH 6.8, 2% SDS, 10% glycerol, 0.1% bromphenol blue, and 5% β-mercaptoethanol) and heated for 5 min at 95°C before SDS-PAGE on 10% gel and subsequently transferred to a pure nitrocellulose membrane. After the transfer, the membrane was blocked with 5% blocking reagent in Tris-buffered saline containing 0.1% Tween-20 overnight at 4°C. The membrane was rinsed in three changes of Tris-buffered saline/Tween-20, incubated in fresh washing buffer once for 15 min and twice for 5 min, and then incubated overnight at 4°C with primary antibodies: rabbit anti-rat DMT1+IRE, DMT1−IRE polyclonal antibodies, rabbit anti-mouse Fpn1 polyclonal antibody (1∶5000); mouse anti-rat TfR1 monoclonal antibody (1∶1000). After three washes, the blots were incubated with goat anti-rabbit or anti-mouse IRDye 800 CW secondary antibody (1∶5000, Li-Cor) for 1 hour at room temperature. The intensity of the specific bands was detected and analyzed by Odyssey infrared imagine system (Li-Cor). To ensure even loading of the samples, the same membrane was probed with rabbit anti-rat β-actin polyclonal antibody at a 1∶2000 dilution.

### Construction of siRNA against DMT-IRE

pSUPER.retro vector (OligoEngine, USA) was used for expression of siRNA targeting on DMT1−IRE. The target sites for siRNA were designed by using an oligoEngine tool. The selected sequences were submitted to a BLAST search to avoid targeting the other human genome. Two 19-nucleotide sequences corresponding to nucleotides 1681–1699 and 1776–1794 of DMT1−IRE were selected to generate the pSUPER-hepcidin vectors. A control vector (pSUPER-Control) was constructed using a 19-nucleotide sequence (gcgcgctttgtaggattcg) with no significant homology to any mammalian gene sequence and therefore served as a non-silencing control (OligoEngine, USA). These sequences were inserted into the pSUPER.retro vector after digestion with BglII and HindIII and were transformed into BL21-A1 One ShotTM supercompetent cells (Invitrogen, USA). Several clones were obtained, and the vectors were amplified. Retroviruses expressing siRNA DMT1−IRE were produced by transfecting pSUPER-hepcidin vectors into amphotropic Phoenix packaging cell line according to the manufacturer's instructions. The obtained retrovirus was used to the in vitro infection of cells or in vivo injection of cerebroventricules.

### Measurement of non-transferrin bound iron uptake

The radio-labelled 55Fe(II) (NTBI) solution was prepared and the Fe(II) uptake was measured as described previously [19]. Following pre-treatment with different concentrations of L-DOPA, the cells were washed with cold PBS and then incubated with or without 55Fe(II) for various periods of time. After being lysed, the cells were scraped off and transferred into Eppendorf tubes. A 50 µl aliquot was subjected to the detection of protein concentration. The cytosol was separated from the stromal fraction by centrifugation at 10,000 g for 20 min at 4°C using a Jouan centrifuge (DJB labcare Ltd., England). Scintillation solution (3 ml) was added to count the cpm. The total iron uptake was the sum of the cytosol and stromal fractions.

### Statistical analysis

Statistical analyses were performed using SPSS software for Windows (version 10.0). Data were presented as mean±SEM. The difference between the means was determined by One-Way ANOVA followed by a Student-Newman-Keuls test for multiple comparisons. A probability value of P<0.05 was taken to be statistically significant.

## Results

### Neurotoxicity induced by L-DOPA: morphological evidence and significantly decreased neuronal viability

**Figure 1:**
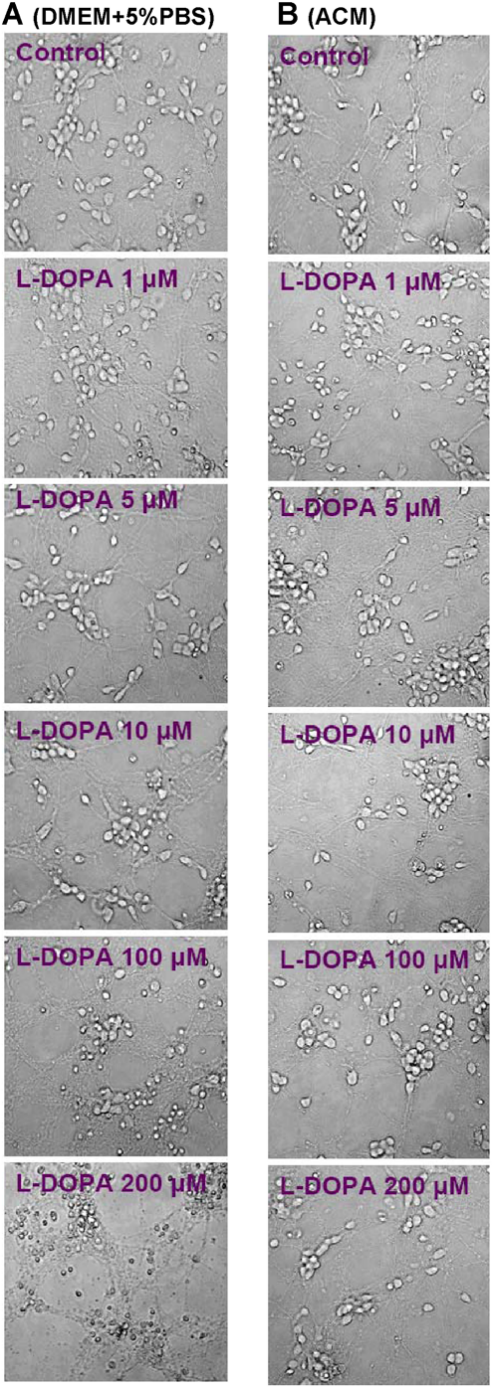
L–DOPA induced morphological changes in cortical neurons. Neurons were treated with L-DOPA (0, 1, 5, 10, 100, 200 µM) in DMEM+5%FBS (A) or astrocyte-conditioned medium (ACM) (B) for 16 hours and then morphological changes were observed as described in Materials and Methods.

**Figure 2:**
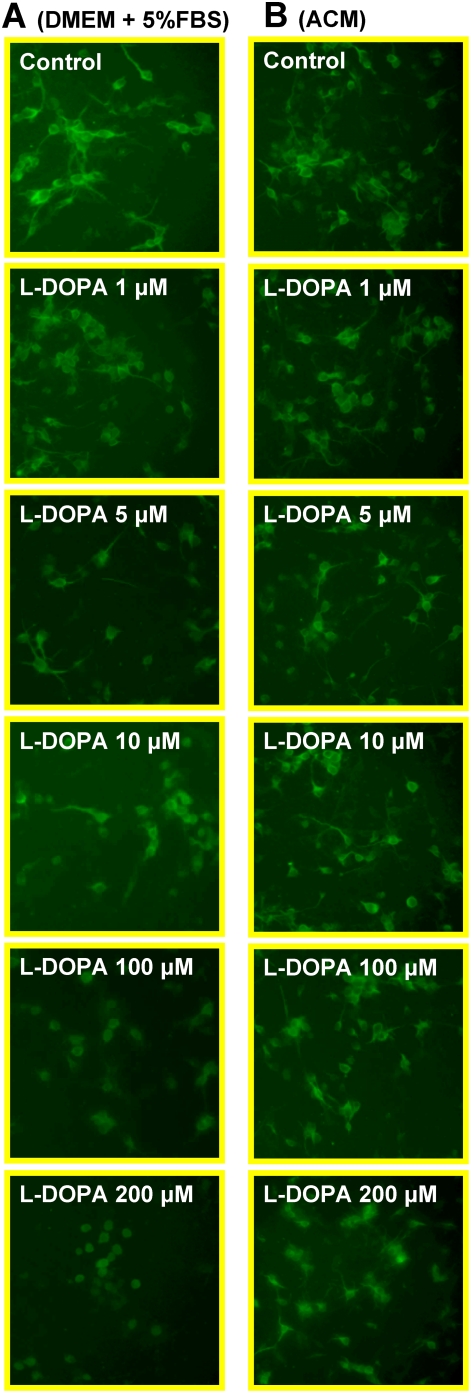
Immunocytochemistry of MAP2 in cortical neurons treated with or without L-DOPA. Neurons were exposed to L-DOPA (0, 1, 5, 10, 100, 200 µM) in DMEM+5%FBS (A) or astrocyte-conditioned medium (ACM) (B) for 16 hours and then immunostained for MAP-2 antibody as described in Materials and Methods.

**Figure 3:**
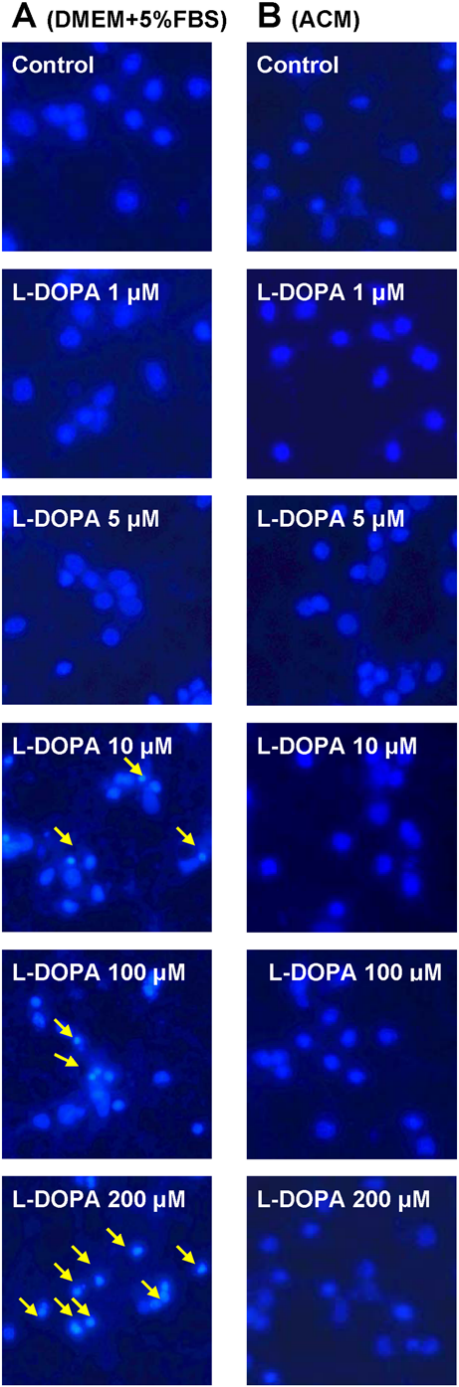
Hoechst 33342 staining in cortical neurons treated with or without L-DOPA. Neurons were exposed to 0, 1, 5, 10, 100 or 200 µM of L-DOPA in DMEM+5%FBS (A) or 0 or 200 µM of L-DOPA in astrocyte-conditioned medium (ACM) (B) for 16 hours and then Hoechst 33342 staining was performed. The nuclei with fluorescing intensely, shrunken in size and irregular in shape are indicated in arrows.

**Figure 4:**
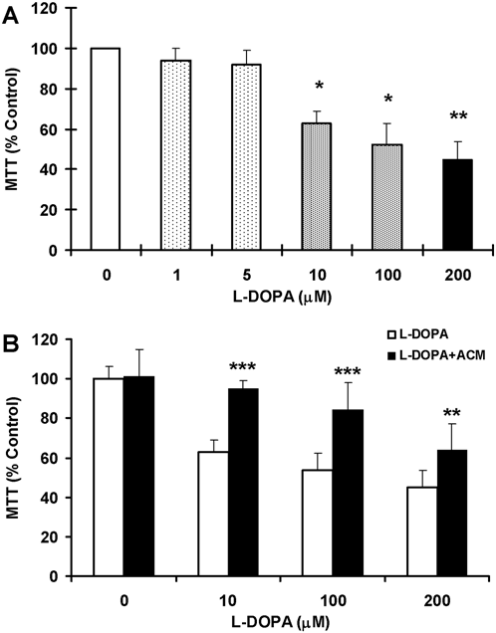
Effects of L-DOPA on neuronal viability. Neurons were treated with 0, 1, 5, 10, 100 or 200 µM of L-DOPA in DMEM+5%FBS for 16 hours (A) or incubated with 0, 10, 100 or 200 µM of L-DOPA in DMEM +5%FBS medium (L-DOPA) or astrocyte-conditioned medium (L-DOPA+ACM) for 16 hours (B). The neuronal viability was then determined using an MTT assay. Data are the mean±SEM (percentage of control) of three independent experiments performed in triplicate. A: *P<0.01, **P<0.001, vs. the control (0 µM). B: **P<0.001, ***P<0.0001 vs. the corresponding controls.

Figure 1A presented the morphological observations in neurons treated with L-DOPA (0, 1, 5, 10, 100 or 200 µM) in DMEM+5%FBS for 16 hours. As compared to the controls, neurons pre-treated with L-DOPA displayed a dose-dependent morphological change. It was characterized by a progressive increase in the number of irregular cell bodies with disrupted and shrunken neuronal processes with the increase in the concentrations of L-DOPA in the medium. Pre-treatment with 200 µM of L-DOPA induced a significant reduction in or disappearance of the neuronal number or processes. The observations under phase contrast microscopy corresponded to the findings of immunostaining of cortical neurons for MAP2 (Figure 2A). Hoechst 33342 staining (Figure 3A) displayed a gradual increase in the number of condensed or fragmented nuclei (apoptosis) in cortical cells with the increase in the concentrations of L-DOPA. The number of condensed nuclei was the highest in the neurons pretreated with 200 µM of L-DOPA. The neuronal viability was determined using an MTT assay. As shown in Figure 4A, treatment of neurons with L-DOPA in DMEM+5%FBS for 16 hours led to a progressive decrease in the cell viability with the increase in the concentrations of L-DOPA. A significant decrease in viability was found in the neurons treated with 10, 100 or 200 µM of L-DOPA (P<0.01 or 0.001).

### ACM diminishes L-DOPA-induced neurotoxicity

Treatment with L-DOPA did not induce any significant changes in neuronal morphology or the immunostaining of cortical neurons for MAP2 when the incubation medium DMEM+5%FBS was replaced by ACM. No significant differences in cell morphology (Figure 1B) or the MAP2 (Figure 2B) were found among the neurons treated with or without different concentrations of L-DOPA. Consistent with this observation, Hoechst 33342 staining demonstrated that cell nuclei remained unchanged in neurons that were incubated with 200 µM of L-DOPA in ACM (Figure 3B). No significant differences in Hoechst 33342 staining were found between the neurons treated with or without 200 µM of L-DOPA in ACM. Furthermore, cell viabilities in the neurons that were incubated with 10, 100 or 200 µM of L-DOPA in ACM were significantly higher than those in the neurons incubated with same concentrations of L-DOPA in DMEM+5%FBS medium (P<0.001 or 0.0001) (Figure 4B).

### L-DOPA induces a significant increase in iron content in cortical neurons

**Figure 5:**
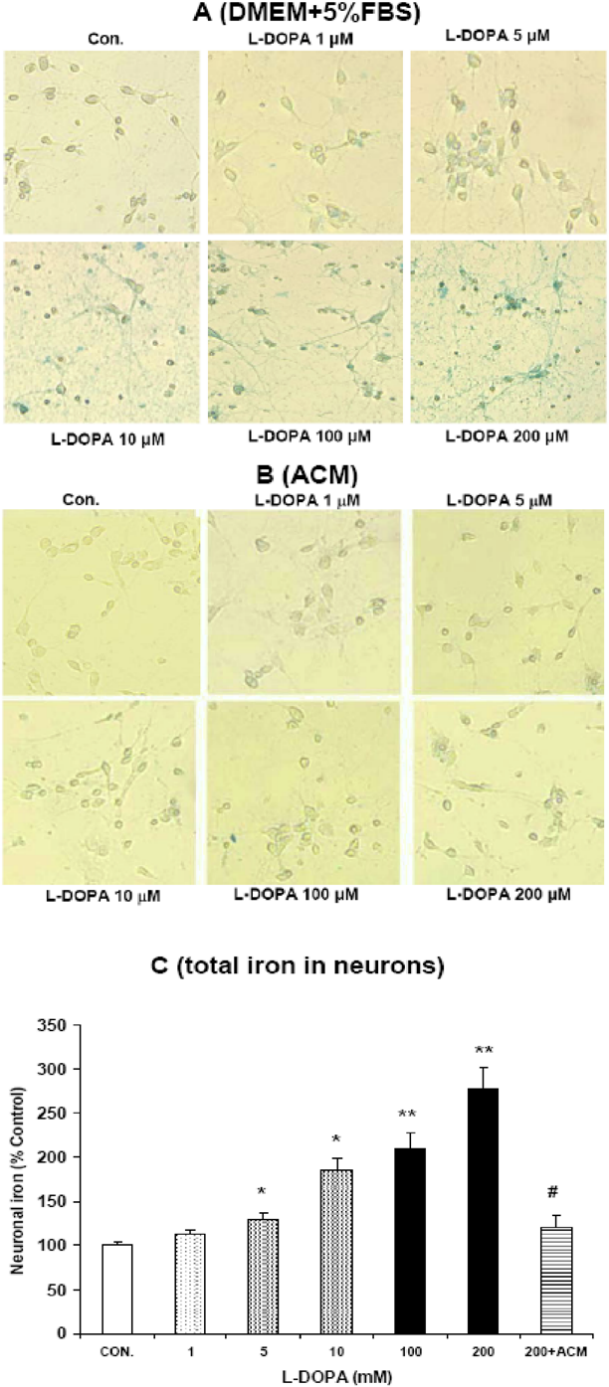
Effects of L-DOPA on neuronal iron. Neurons were exposed to L-DOPA (0, 1, 5, 10, 100, 200 µM) in DMEM+5%FBS (A) or astrocyte-conditioned medium (ACM) (B) for 16 hours and then iron staining was conducted. Total iron was also measured using a GFAAS method in the neurons treated with or without different concentrations of L-DOPA in the presence or absence of ACM (C). Data are the mean±SEM (percentage of control) of three independent experiments performed in triplicate. *P<0.05, **P<0.01 vs. the control (0 µM); #<0.01 vs. 200 µM of L-DOPA.

Figure 5A presented the data on iron stain in neurons that were treated with different concentrations of L-DOPA in DMEM+5%FBS for 16 hours. The control neurons had large cell bodies and more elaborate processes with a tiny amount of iron located primarily in the cell bodies. No changes in iron distribution and morphology were found in neurons pre-treated with 1 µM of L-DOPA. In the neurons treated with 5 µM of L-DOPA, iron content in the cell body appeared to have increased but the body shape and body size remained the same. A significant increase in iron density and positive iron staining in the processes was observed in the neurons treated with 10, 100 or 200 µM of L-DOPA. The iron accumulation was most pronounced in neurons pre-treated with 200 µM of L-DOPA, which appeared dystrophic with large and irregularly-shaped cell bodies attaching to densely beaded processes and the iron filling the cell body and processes completely. The measurement of total iron provided quantitative evidence for the ability of L-DOPA to induce a significant increase in neuronal iron contents. The total iron was significantly higher in neurons pretreated with 5, 10, 100 or 200 µM of L-DOPA than in the controls (Figure 5C).

### ACM reduces iron content in cortical neuron treated with L-DOPA

Figure 5B presented the data on iron stain in neurons that were treated with different concentrations of L-DOPA in ACM. No significant changes in neuron iron contents were observed when neurons were incubated in ACM with L-DOPA (0, 1, 5, 10, 100 or 200 µM) for 16 hours (Figures 5B). Treatment with ACM induced a significant decrease in iron content in cortical neurons treated with 200 µM of L-DOPA (Figure 5C). These results demonstrated that ACM has the ability to inhibit the effect of L-DOPA on iron content in cortical neurons.

### L-DOPA induces a significant increase in DMT1−IRE expression and ferrous iron uptake in cortical neurons

**Figure 6:**
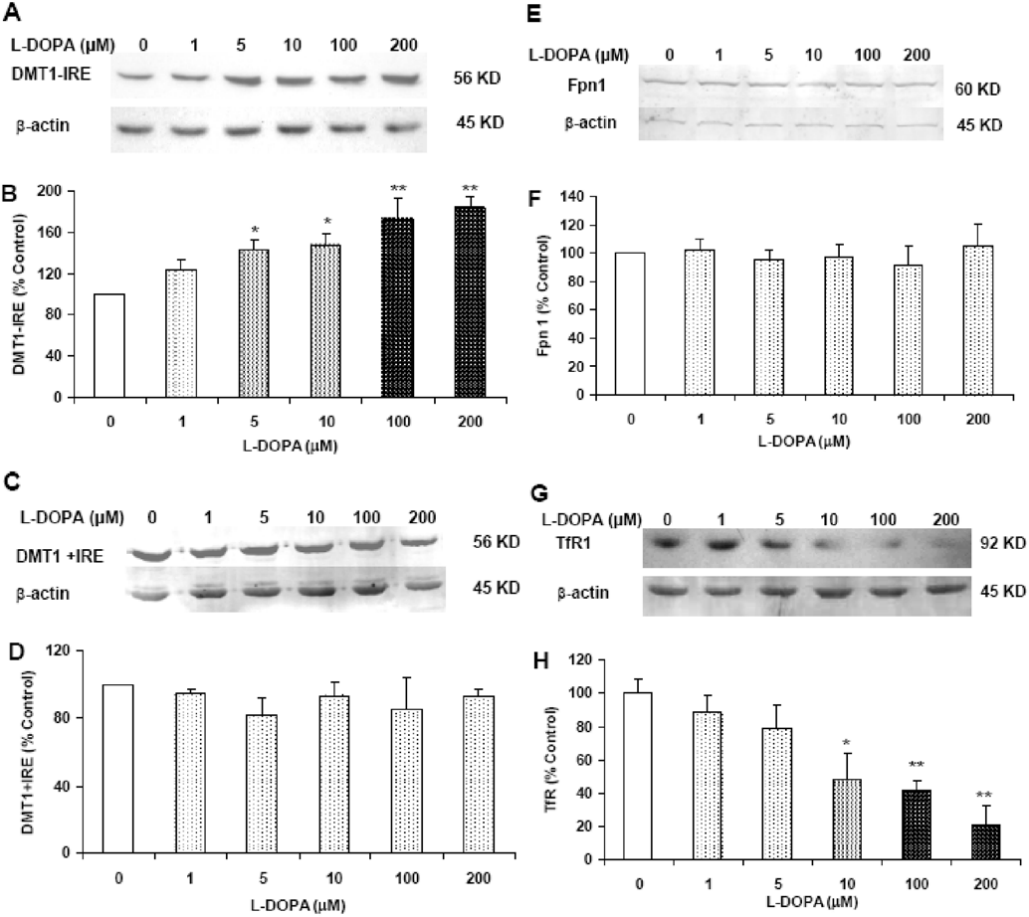
Effects of L-DOPA on the expression of DMT1−IRE, DMT1+IRE, Fpn1 and TfR1 proteins in cortical neurons. Neurons were treated with L-DOPA (0, 1, 5, 10, 100, 200 µM) in DMEM+5%FBS for 16 hours and the expression of proteins was determined using Western blot analysis. A, representative Western blots of DMT1−IRE and β-actin. B, the relative values of DMT1−IRE protein expression. C, representative Western blots of DMT1+IRE and β-actin. D, the relative values of DMT1+IRE protein expression. E, representative Western blots of Fpn1 and β-actin. F, the relative values of Fpn1 protein expression. G, representative Western blots of TfR1 and β-actin. H, the relative values of TfR1 protein expression. Data are means±SEM (percentage of control) of four independent experiments. L-DOPA treatment induced a dose-dependent decrease in TfR. There were no significant differences in the levels of DMT1+IRE and Fpn1 protein between neurons treated with and without L-DOPA. * P<0.05, ** P<0.01 vs. the control.

**Figure 7:**
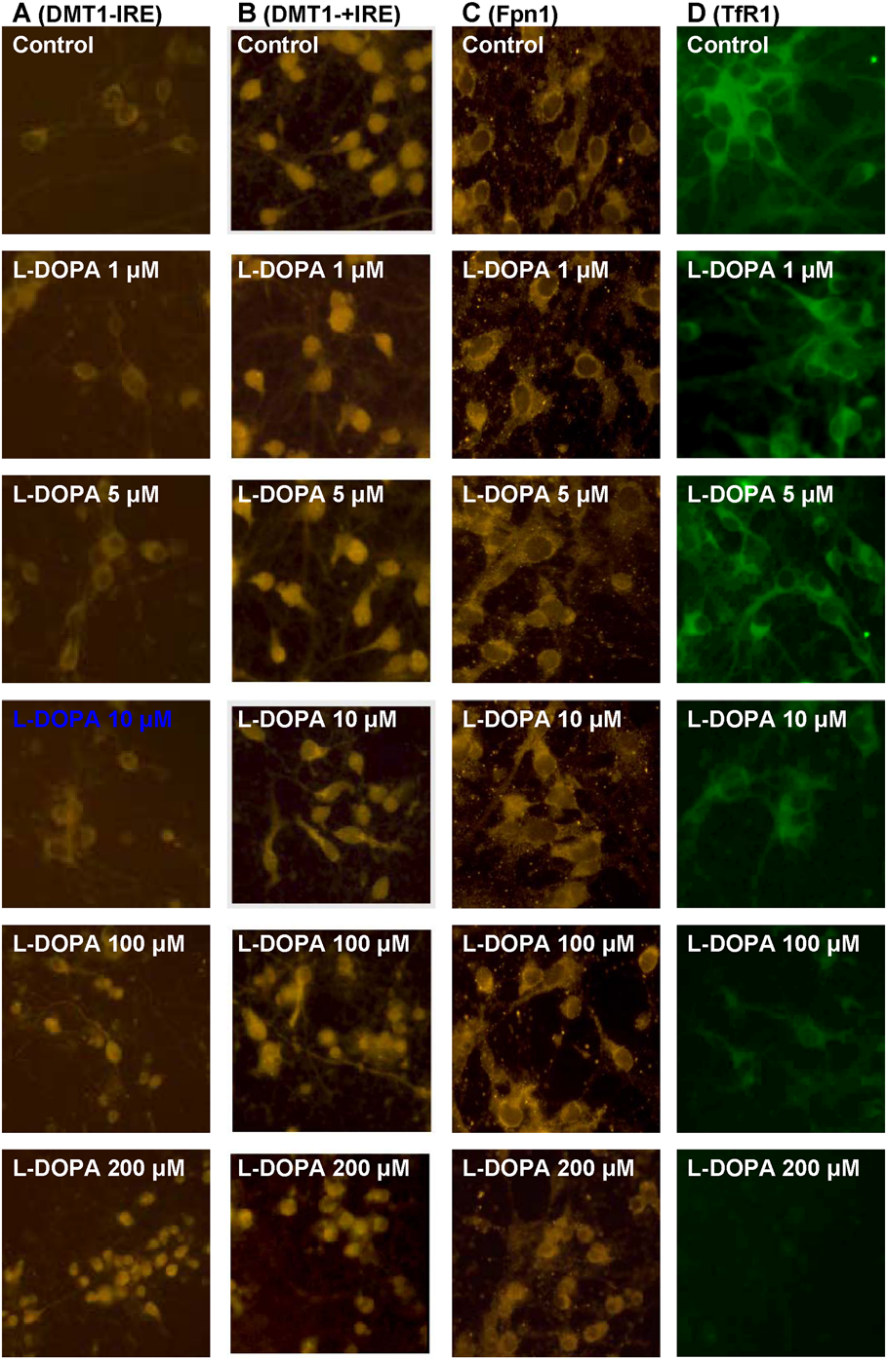
Immmocytochemistry of DMT1−IRE, DMT1+IRE, Fpn1 and TfR1 proteins in cortical neurons. Cells were treated with L-DOPA (0, 1, 5, 10, 100, 200 µM) in DMEM+5%FBS for 16 hours and then immunostained for DMT1−IRE (A), DMT1+IRE (B), Fpn1 (C) or TfR1 (D) antibodies. L-DOPA treatment induced a dose-dependent decrease in TfR1 expression, but no significant changes were observed in the expression of DMT1+IRE and Fpn1 proteins.

L-DOPA treatment induced a significant and dose-dependent increase in the expression of DMT1−IRE (Figures 6A,B). The levels of DMT1−IRE in the neurons pre-treated with 5, 10, 100 or 200 µM of L-DOPA were significantly higher than those in the controls (P<0.05 or 0.01). The results from immunostaining of DMT1 also indicated that L-DOPA has a significant ability to increase the expression of DMT1−IRE rather than DMT1+IRE in cortical neurons (Figure 7A). However, treatment of cortical neurons with L-DOPA did not induce any changes in the expression of DMT1+IRE (Figures 6C,D and Figure 7B) or Fpn1 (Figures 6E,F and Figure 7C). This implies that the increased iron contents in the neurons pre-treated with L-DOPA are associated neither with DMT1+IRE nor the decreased Fpn1-mediated iron release from the cells. Western blot analysis also demonstrated that treatment with L-DOPA induces a dose-dependent decrease in TfR1 (Figures 6G,H and Figure 7D), rather than an increase as we expected. This shows that TfR1 is not involved in the increased neuronal iron contents induced by L-DOPA.

**Figure 8:**
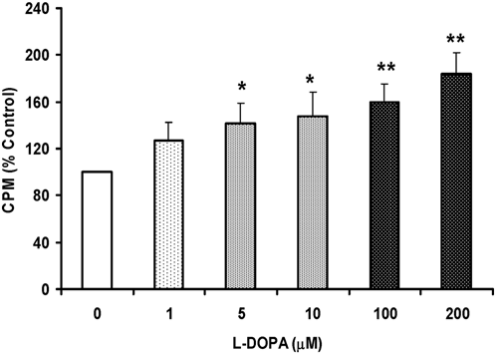
Effects of L-DOPA on 55Fe(II) uptake by cortical cells. Neurons were treated with L-DOPA (0, 1, 5, 10, 100, 200 µM) in DMEM+5%FBS for 16 hours and then incubated with 2 µM of 55Fe(II) in 0.27 M sucrose (pH 6.5) at 37°C for 30 min. The 55Fe(II) taken into neurons was then measured. Data were presented as mean±SEM of 6 independent experiments performed in triplicate. *P<0.05, **P<0.01 vs. the control.

These findings led us to hypothesize that the increased DMT1−IRE expression might induce an increase in DMT1−IRE-mediated ferrous iron uptake by neurons. This might be one of the reasons for the L-DOPA-induced increase in neuronal iron contents. Therefore, we then examined effects of L-DOPA on ferrous iron uptake in neurons. As shown in Figure 8, treatment with L-DOPA induced a significant and dose-dependent increase in the 55Fe uptake by neurons. The 55Fe uptake was significantly higher in the neurons pre-treated with 5, 10, 100 or 200 µM of L-DOPA than those in the controls (P<0.05 or 0.01). Correlation analysis of the relationship between the expression of DMT1−IRE protein and the 55Fe uptake in the neurons was conducted by plotting the values for these two indicators against one another. A highly significant correlation was found in the cells pre-treated with different concentrations of L-DOPA (Y = 0.924x+11.603, R2 = 0.974, P<0.001). The relationship between the expression of DMT1−IRE protein and neuronal viability was also determined by the same analysis. A highly significant correlation was also found in the cells pre-treated with different concentrations of L-DOPA (Y = −0.7165x+178.28, R2 = 0.853, P<0.01).

### ACM reduces DMT1−IRE expression in cortical neuron treated with L-DOPA

**Figure 9:**
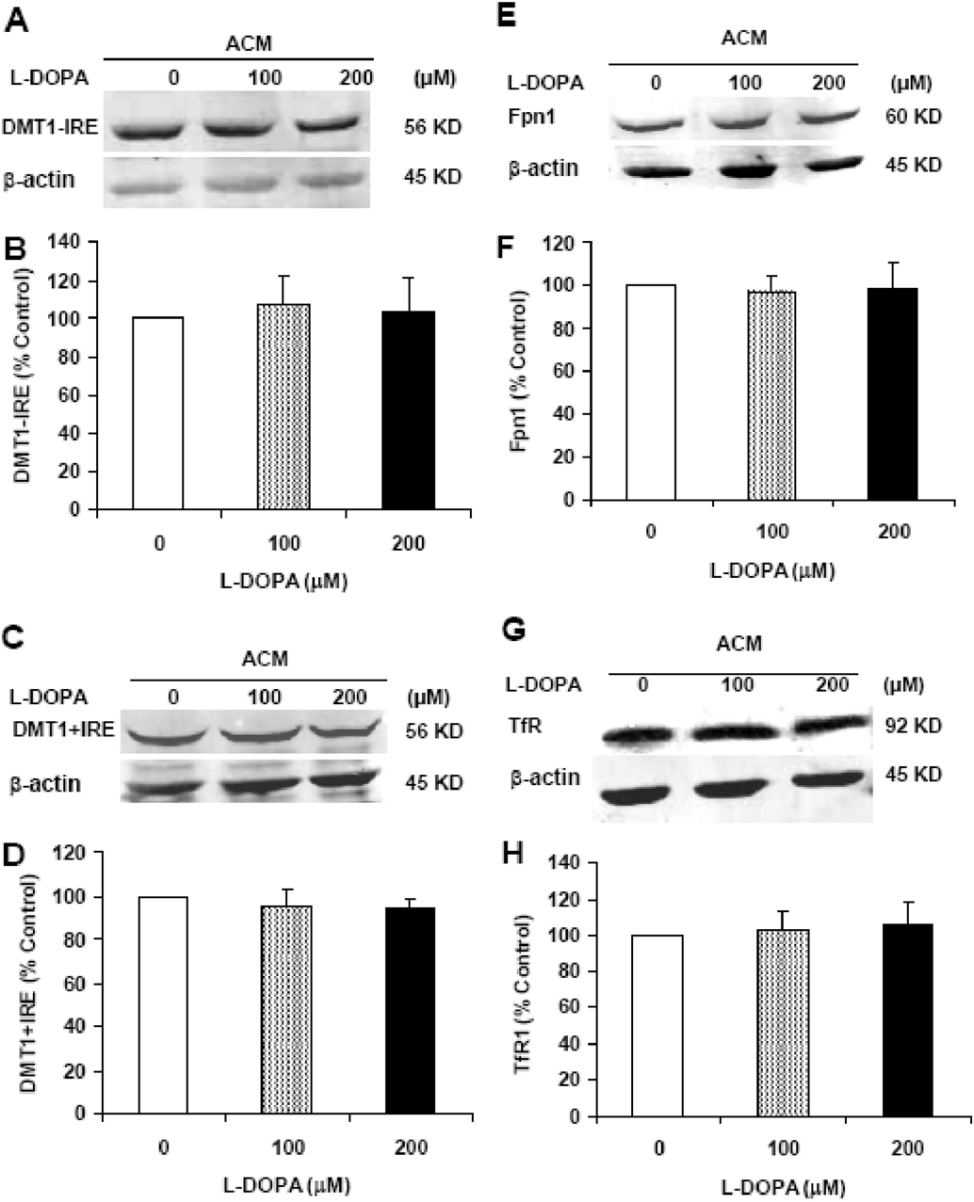
Effects of astrocyte-conditioned medium on the expression of DMT1−IRE, DMT1+IRE, Fpn1 and TfR1 proteins in cortical neurons treated with L-DOPA. Neurons were treated with L-DOPA (0, 100 or 200 µM) in ACM for 16 hours and Western blot analysis was then conducted. A, representative Western blots of DMT1−IRE and β-actin. B, the relative values of DMT1−IRE protein expression. C, representative Western blots of DMT1+IRE and β-actin. D, the relative values of DMT1+IRE protein expression. E, representative Western blots of Fpn1 and β-actin. F, the relative values of Fpn1 protein expression. G, representative Western blots of TfR1 and β-actin. H, the relative values of TfR1 protein expression. Data are means±SEM (percentage of control) of four independent experiments. There were no significant differences in the levels of all four proteins we investigated between the neurons treated with or without L-DOPA.

No significant differences were found in the expression of DMT1−IRE (Figures 9A,B) or other iron transport proteins including DMT1+IRE (Figures 9C,D), Fpn1 (Figures 9E,F) and TfR1 (Figures 9G,H) among the neurons incubated in ACM with 0, 100 or 200 µM of L-DOPA. The data imply that ACM has a role to inhibit the increased expression of DMT1−IRE induced by L-DOPA in cortical neurons. Although we did not measure ferrous iron uptake in the cortical neurons treated with 0, 100 or 200 µM of L-DOPA in the ACM media, it is reasonable to believe that ACM also has the ability to inhibit the increased ferrous iron uptake induced by L-DOPA in cortical neurons. Thus, the significant role of ACM in protecting cortical neurons from L-DOPA toxicity is “DMT1−IRE and iron”-mediated. To find out whether antioxidant has the same effect as ACM, we also investigated the effects of antioxidant on L-DOPA induced-neurotoxicity by the addition of different doses of superoxide dismutase (SOD) to the neuron culture medium. It was found that SOD has a significant role to protect neurons from L-DOPA-induced injury (data not shown) which suggested that the neuroprotective role of ACM is also partly associated with its antioxidative acticity which might be due to the antioxidants supplied by astrocytes.

### Decreased expression of DMT1−IRE induced by siRNA DMT-IRE reduces L-DOPA neurotoxicity

**Figure 10:**
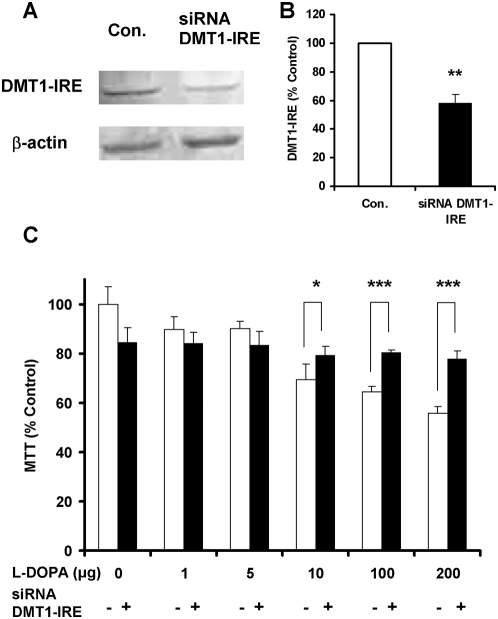
Effects of siRNA DMT−IRE on DMT1−IRE expression and L-DOPA-induced neurontoxicity in cortical neurons. A and B: Neurons were pre-incubated with siRNA DMT−IRE for 24 hours and Western blots analysis was then performed (A: representative Western blots of DMT1−IRE and β-actin, and B: the relative values of DMT1−IRE protein in neurons infected with or without siRNA DMT−IRE). Data are means±SEM (percentage of control) of four independent experiments performed in triplicate. **P<0.01 vs. the control. C: Neurons were pre-incubated with siRNA DMT−IRE for 24 hours and then treated with different concentrations of L-DOPA (0, 1, 5, 10, 100, 200 µM) in DMEM+5%FBS medium fro 16 hours. The neuronal viability was then determined using an MTT assay. Data are the mean±SEM (percentage of control) of four independent experiments performed in triplicate. *P<0.05, ***P<0.001 vs. the corresponding controls.

We then investigated the effects of siRNA DMT−IRE on DMT1−IRE expression and L-DOPA-induced neurontoxicity in cortical neurons. Incubation of neurons with siRNA DMT−IRE for 24 hours induced a significant decrease in the expression level of DMT1−IRE protein in the infected neurons (Figures 10A and B). When neurons were pre-incubated with siRNA DMT−IRE for 24 hours and then treated with different concentrations of L-DOPA for 16 hours, it was found that the viabilities of the neurons infected with ‘siRNA DMT−IRE’ were significantly higher than those of the control neurons at 10, 100 or 200 µg of L-DOPA (p<0.05, 0.01 or 0.01) (Figure 10C). The findings demonstrated that the decreased expression of DMT1−IRE induced by siRNA DMT−IRE can reduce L-DOPA neurotoxicity.

## Discussion

The exact etiology of the neurotoxicity induced by L-DOPA is unknown although there is evidence that abnormal pulsatile stimulation of dopamine receptors may be contributory to it [20], [21]. Based on our recent findings [10], together with the potential role of DMT1−IRE in neuronal iron uptake [10], [22] and the well-known function of iron in the generation of reactive oxygen species, we hypothesized that the upregulation of DMT1−IRE might play a critical role in the development of L-DOPA neurotoxicity. In the present study, efforts were made to test this hypothesis. Based on several lines of solid evidence obtained from this study, we concluded that the L-DOPA-induced neurotoxicity is mediated by the upregulation of DMT1−IRE expression in cortical neurons.

The first evidence comes from the investigation of the effects of L-DOPA on cortical neurons. Our data clearly demonstrated that treating neurons with different concentrations of L-DOPA induces not only a significant change in morphology or Hoechst-33342 staining but also a dose-dependent decrease in neuronal viability, confirming that L-DOPA can induce neurotoxicity in our experimental conditions. Meanwhile, the treatment also resulted in a significant dose-dependent increase in iron content, ferrous iron uptake and DMT1−IRE protein expression in cortical neurons. Furthermore, correlation analysis showed that the expression of DMT1−IRE protein is significantly correlated with 55Fe uptake (positively) and neuronal viability (negatively) in cortical neurons treated with different concentrations of L-DOPA; respectively. These findings support a role of L-DOPA in the increased DMT1−IRE expression as well as in the connection of the increased DMT1−IRE with the increased neuron iron content. In addition, both western blot and immunocytochemistry analysis showed that L-DOPA didn't induce any changes in the expression of DMT1+IRE or Fpn1. A significant decrease in TfR1 expression, rather than an increase, was found in the neurons treated with L-DOPA. These results showed that the increase in neuronal iron content induced by L-DOPA is due to neither the decrease in Fpn1-mediated iron efflux nor the increase in DMT1+IRE or TfR1-mediated iron uptake. On the other hand, these results also provided further support that L-DOPA has a role to increase DMT1−IRE expression and that the increased iron content in neurons induced by L-DOPA results from the increased DMT1−IRE-mediated iron uptake.

The second evidence is obtained from the study on the effects of ACM on L-DOPA-induced neurotoxicity. It has been reported that astrocytes have a role in the reduction of neuronal cell death following a variety of cellular stresses, such as excitotoxicity and oxidative stress [23]–[25]. Also, it has been demonstrated that glia conditioned medium (GCM) protects fetal rat midbrain neurons in culture from L-DOPA toxicity [26]. We speculated that the neuroprotective role of GCM or ACM might be mediated by their ability to inhibit DMT1−IRE expression as well as iron accumulation in neurons. Therefore, we investigated the effects of ACM on morphology, Hoechst 33342 staining, viability, neuron iron content and DMT1−IRE expression in the neurons treated with L-DOPA by incubating neurons in ACM with or without different concentrations of L-DOPA for 16 hours. The results obtained on the morphology, Hoechst 33342 staining and neuronal viability clearly demonstrated that ACM significantly diminishes L-DOPA-induced neurotoxicity. No significant differences were found in morphology, Hoechst-33342 staining and viability between the neurons incubated in ACM in the presence and those incubated in ACM in the absence of the same concentrations of L-DOPA. At the same time, western blot analysis and iron staining showed that ACM significantly reduces DMT1−IRE expression as well as neuron iron content. The similar tendencies of the effects of ACM on L-DOPA-induced neurotoxicity, DMT1−IRE expression and neuron iron content implied that ACM protects neurons from L-DOPA by its ability to inhibit DMT1−IRE expression and ferrous iron uptake.

The final piece of evidence is provided by the data on the effects of siRNA DMT−IRE on L-DOPA neurotoxicity. According to the findings on DMT1−IRE, L-DOPA neurotoxicity and the role of ACM, we expected that a decrease in DMT1−IRE expression should also be able to lead to a decrease in L-DOPA neurotoxicity. Therefore, we infected the cortical neurons with siRNA DMT−IRE to decrease DMT1−IRE expression to see whether L-DOPA neurotoxicity was hence changed in these neurons. As we expected, western blot analysis revealed a significant decrease in the expression of DMT1−IRE protein as well as iron content in the neurons infected with siRNA DMT−IRE. The MTT assay demonstrated that the viabilities in the neurons infected with siRNA DMT−IRE were significantly higher than those in the control neurons at all concentration points of L-DOPA we examined. The findings showed that the decreased expression of DMT1−IRE induced by siRNA DMT−IRE can decrease neuronal iron accumulation and also L-DOPA neurotoxicity. These further confirmed that DMT1−IRE upregulation and the increased DMT1−IRE mediated iron influx play a key role in the development of L-DOPA neurotoxicity in primary neurons.

DMT1 (DCT1 or NRAMP2), a widely expressed membrane protein [27], [28], is responsible for the uptake of a broad range of divalent metal ions [27]–[29]. There are four DMT1 isoforms that differ in their N- and C-termini arise from mRNA transcripts that vary both at their 5′-ends (starting in exon 1A or exon 1B) and at their 3′-ends giving rise to mRNAs containing (+) or lacking (−) the 3′-IRE [30]. The existence of DMT1 in the brain has been well determined [17], [31]. The functions of DMT1+IRE and DMT1−IRE have not been completely understood [32]. However, available data support the notion that DMT1−IRE, rather than DMT1+IRE, is the entity responsible for the transmembrane transport of iron released from transferrin to the early endosomal lumen [10], [22]. Recent studies also inferred that DMT1−IRE is predominantly found in three compartments: the plasma membrane, early/recycling endosomes, and the endoplasmic reticulum [22]. In the present study, we found that treatment of neurons with different concentrations of L-DOPA also resulted in a significantly dose-dependent increase in iron content, ferrous iron uptake and the expression of DMT1−IRE protein in cortical neurons. It was also found that there is a significant correlational relationship between the expression of DMT1−IRE protein and 55Fe uptake (positively) in cortical neurons treated with different concentrations of L-DOPA. These findings provide further support to the notion that DMT1−IRE, rather than DMT1+IRE, is the entity responsible for iron transmembrane transport as well as the role of DMT1−IRE upregulation in L-DOPA neurotoxicity.

In summary, in the present study we provided solid evidence for the first time for the association of DMT1−IRE with neurotoxicity induced by L-DOPA. We concluded that upregulation of DMT1−IRE and then the increased DMT1−IRE mediated iron influx play a critical role in L-DOPA neurotoxicity in primary cortical neurons. We also demonstrated for the first time that ACM's protection of cortical neurons from L-DOPA is at least partly due to its ability to inhibit DMT1−IRE expression and ferrous iron uptake and its antioxidative activity which might be due to the antioxidants supplied by astrocytes.
